# Fractal Correlation Properties of Heart Rate Variability: A New Biomarker for Intensity Distribution in Endurance Exercise and Training Prescription?

**DOI:** 10.3389/fphys.2020.550572

**Published:** 2020-09-18

**Authors:** Thomas Gronwald, Bruce Rogers, Olaf Hoos

**Affiliations:** ^1^Department of Performance, Neuroscience, Therapy and Health, Faculty of Health Sciences, MSH Medical School Hamburg, University of Applied Sciences and Medical University, Hamburg, Germany; ^2^Department of Internal Medicine, College of Medicine, University of Central Florida, Orlando, FL, United States; ^3^Center for Sports and Physical Education, Julius Maximilians University of Würzburg, Würzburg, Germany

**Keywords:** autonomic nervous system, HRV, detrended fluctuation analysis, intensity distribution, intensity zones, endurance exercise, endurance training, polarized training

## Abstract

Exercise and training prescription in endurance-type sports has a strong theoretical background with various practical applications based on threshold concepts. Given the challenges and pitfalls of determining individual training zones on the basis of subsystem indicators (e.g., blood lactate concentration, respiratory parameters), the question arises whether there are alternatives for intensity distribution demarcation. Considering that training in a low intensity zone substantially contributes to the performance outcome of endurance athletes and exceeding intensity targets based on a misleading aerobic threshold can lead to negative performance and recovery effects, it would be desirable to find a parameter that could be derived via non-invasive, low cost and commonly available wearable devices. In this regard, analytics conducted from non-linear dynamics of heart rate variability (HRV) have been adapted to gain further insights into the complex cardiovascular regulation during endurance-type exercise. Considering the reciprocal antagonistic behavior and the interaction of the sympathetic and parasympathetic branch of the autonomic nervous system from low to high exercise intensities, it may be promising to use an approach that utilizes information about the regulation quality of the organismic system to determine training-intensity distribution. Detrended fluctuation analysis of HRV and its short-term scaling exponent alpha1 (DFA-alpha1) seems suitable for applied sport-specific settings including exercise from low to high intensities. DFA-alpha1 may be taken as an indicator for exercise prescription and intensity distribution monitoring in endurance-type sports. The present perspective illustrates the potential of DFA-alpha1 for diagnostic and monitoring purposes as a “global” system parameter and proxy for organismic demands.

## 1. Introduction: Exercise Prescription and Intensity Distribution for Endurance Exercise and Training

Endurance exercise and training may encompass a wide spectrum of workloads, from many hours spent at low exertion to brief high intensity intervals lasting several seconds. These efforts are typically separated into 3 intensity zones based on certain physiologic thresholds. The first to second intensity zone boundary is generally defined by the first lactate (LT1) or ventilatory threshold (VT1) as an aerobic threshold (Meyer et al., 2005; Beneke et al., 2011; Hofmann and Tschakert, 2017; Bourgois et al., 2019). The second to third intensity zone transition is felt to be related to the second lactate threshold (LT2), maximum lactate steady state (MLSS), second ventilatory threshold (VT2), or respiratory compensation point (RCP) as an anaerobic threshold (Bourgois et al., 2019). Accurate and easy determinations of these thresholds would therefore be essential to set up and compare the various training-intensity distribution strategies for fitness optimization and performance enhancement as well as for mathematical modeling of zone training time or distance parameters (Seiler and Kjerland, 2006; Treff et al., 2019). Failure to have consistent definition of these limits could lead to undesirable training loads and distributions within the different training zones.

Studies have attempted to show both the advantages and adverse consequences of time spent training in the various zones. Training with large quantities of low intensity exercise seems to relate to future success and performance in the half and full marathon. The volume of “easy runs” done by competitive long-distance runners (Casado et al., 2019) as well as a high training volume done by recreational half marathon runners (Fokkema et al., 2020) was related to their future performance. Interestingly, in a study of sedentary men and women, cardiac structural remodeling began to occur during the initial months of low intensity endurance training volume, before the onset of high intensity interval work (Arbab-Zadeh et al., 2014). Both high intensity training (Wen et al., 2019) as well as combining low and high intensity training (Laursen, 2010; Pugliese et al., 2018) have been shown to be advantageous to different aspects of exercise performance. For the accurate quantification of training-intensity distributions, various models have been employed, such as polarized, threshold, or pyramidal training-intensity distribution (Seiler and Kjerland, 2006; Esteve-Lanao et al., 2007; Stöggl and Sperlich, 2015, 2019; Bourgois et al., 2019). Although incremental exercise tests with measurement of lactate concentration and/or gas exchange are commonly done to define thresholds, the different approaches may not agree, leading to inaccurate delineation of zone boundaries (Chicharro et al., 1997; Hopker et al., 2011; Pallarés et al., 2016). This can result in misleading comparisons between protocols as well as undesired training outcomes in athletes attempting to emulate a proposed method. The physical sequelae of even minimally exceeding intensity targets may include glycogen depletion (Beneke et al., 2011), prolonged cardiac parasympathetic recovery (Seiler et al., 2007; Stanley et al., 2013), gastrointestinal barrier disruption (van Wijck et al., 2012) along with more overall central and muscular fatigue (Noakes et al., 2005; Venhorst et al., 2018).

In order to study changes in physiological responses and performance outcomes after training zone ratio manipulation, there is a desperate need for an agreement on what constitutes threshold boundary markers. The body of literature for zone 1 to zone 2 transition seems particularly confusing (Faude et al., 2009; Mann et al., 2013; Hall et al., 2016). In a study looking at time spent in each training zone with heart rate and speed defined by gas exchange parameters, a greater fraction of relative time was spent in zone 2 if the zone was defined by a subjects heart rate rather than running speed (Bellinger et al., 2019). Of importance is also the observation that both VT1 and VT2 do not correlate well with fixed percentages of either maximum heart rate, VO_2MAX_ or maximum aerobic power in diverse populations (Peiffer et al., 2008; Azevedo et al., 2011; Hansen et al., 2019; Iannetta et al., 2020), making training recommendations based on these pre-set metrics problematic. Evaluating performance outcomes using fixed lactate values (Stöggl and Sperlich, 2014) or different measures of LT1/VT1 (Esteve-Lanao et al., 2007; Muñoz et al., 2014; Manunzio et al., 2016) have also led to inconsistent results as their comparison is difficult for the following reasons. Published studies show disagreement in calculated LT1 work rates based on log-log or linear spline intersection methods (Newell et al., 2007; Faude et al., 2009; Jamnick et al., 2018). Other investigators used either a fixed amount of lactate rise from baseline (1 mmol/l) or a fixed value of 2 mmol/l which yielded diverse exercise intensities (Faude et al., 2009; Jamnick et al., 2018). Importantly, a protocol using a specific lactate cut-off of 2 mmol/l as a low intensity marker may be tolerated differently, for example by men vs. women. During an incremental test, men reached a fixed lactate of 2 mmol/l at lower relative intensities than women despite both sexes having comparative log-log derived LT1 (Sargent and Scroop, 2007). In regards to gas exchange testing, the derived VT1 value is not without potential error (Vainshelboim et al., 2017). Metabolic cart software driven calculation of VT1 may be subject to significant variation (Ekkekakis et al., 2008). Visual estimation accuracy based on the V-slope technique or PetO2 is dependent on user experience (Yeh et al., 1983) and inter-observer variability (Meyer et al., 1996). Up to 10% of gas exchange tests may have an indeterminate VT1 despite employing the triple detection method (Gaskill et al., 2001). Interestingly, even though lactate and ventilatory thresholds are generally felt to be related to the same physiologic phenomena, correlation between the two is subject to substantial variation (Pallarés et al., 2016). Whether this is related to underlying differences in biologic mechanism or simply from protocol variation is unclear (Plowman and Smith, 2017). Finally, both gas exchange and lactate testing strongly depend on exercise test protocol, which may or may not produce threshold values equivalent to constant load exercise (Iannetta et al., 2019). Even if correction methods are applied, transfer of results measured by laboratory treadmill or cycle ergometry to actual trail running, skiing or road cycling may be altered through changes in biomechanical efficiency (Beneke et al., 2011).

Given the above mentioned difficulties in defining markers for zone 1 transition, the question arises whether there are alternatives for intensity distribution demarcation. Training in zone 1 substantially contributes to the performance outcome of endurance athletes (e.g., for polarized training-intensity distribution: zone 1−70 to 80%, zone 2−0 to 10% and zone 3—up to 20% of total training volume, Seiler and Kjerland, 2006), and exceeding intensity targets based on a misleading aerobic threshold can lead to potentially negative performance and recovery effects (Stanley et al., 2013). Even more desirable would be a parameter that could be derived via non-invasive, low cost and commonly available wearable devices (Düking et al., 2016). One of the easy accessible subjective variables is the rating of perceived exertion (RPE), which has proven as sensitive for evaluating organismic system fatigue during exercise (Eston, 2012), but differs regarding results of training-intensity distribution from other methods (Bellinger et al., 2019). In addition, various indexes of heart rate variability (HRV; providing heart rate time series by RR-intervals and refers to the potential changing patterns of cardiac beat to beat timing, which after statistical analysis may provide physiologic information on autonomic nervous system outflow on the heart; Billman, 2011) resulting from time- and frequency-domain analysis have been studied during dynamic exercise and have been shown to alter as work rates increase, with the greatest change occurring during lower intensities (Tulppo et al., 1996; Sandercock and Brodie, 2006; Karapetian et al., 2008; Michael et al., 2017). Despite well-functioning approaches (Tulppo et al., 1996; Karapetian et al., 2008), frequency-domain parameters such as high frequency (HF) power for example has been noted to be unreliable in a sizable fraction of individuals with up to 20% of subjects not having identified breakpoints (Cottin et al., 2006). Furthermore, Blasco-Lafarga et al. (2017) noted that standard deviation 1 from Poincaré plot analysis (SD1, short-term) was already suppressed in young athletes at the first tested work rate of 60% VO_2MAX_ making it less useful for example for aerobic threshold delineation. However, in response to the concern over strongly decreased variability and weak reproducibility of amplitude dependent time- and frequency-domain HRV measures during exercise (Persson and Wagner, 1996; Tulppo et al., 2005), non-linear methods of HRV analysis reveal promising approaches gaining new insights for training-intensity distribution from a holistic autonomic nervous system (ANS) perspective, not depending on specific organismic subsystems.

## 2. Correlation Properties of HRV

In the last decade, analytics conducted from non-linear dynamics of HR time series have been adapted to gain further insights into the complex cardiovascular regulation during endurance-type exercise of different types and modes (Hottenrott and Hoos, 2017; Michael et al., 2017; Gronwald and Hoos, 2020). Thus, certain measures of HR time series may provide new opportunities for diagnostic and monitoring of cardiac autonomic regulation. Physiologic research suggests that cardiac dynamics are controlled by complex interaction effects between the sympathetic and parasympathetic branch of the ANS on the sinus node, and non-neural factors (Persson, 1996; Qu et al., 2014). During exercise, these two branches interact in a reciprocal antagonist way, resulting in a gradient of response between sympathetic activation and parasympathetic withdrawal depending on exercise intensity (Sandercock and Brodie, 2006; White and Raven, 2014; Michael et al., 2017). Considering the reciprocal antagonistic behavior and the interaction of the two branches of the ANS from low to high exercise intensities, it may be promising to use an approach that utilizes information about the regulation quality of the organismic system during exercise to determine training-intensity distribution on that basis.

The general background of this rationale is that the HRV signal consists not only of quasi-periodic oscillations, but it also possesses random fluctuations and fractal structures (Goldberger et al., 2002). Analysis of these structures were already conducted in the investigation of age and disease regarding qualitative characteristics of the structure (scaling), dynamics of the signal, and interaction of involved subsystems (Mansier et al., 1996; Aubert et al., 2003; Voss et al., 2009). One widely applied approach to analyse such characteristics is the non-linear method of detrended fluctuation analysis (DFA) with the possibility to get more insights into the correlation properties of HR time series caused by physiological processes (Goldberger et al., 2002; Hottenrott and Hoos, 2017). As a modification of the root mean square analysis (RMS) the DFA showed a low dependence on HR and is also suitable for investigating short and non-stationary data of time series (Peng et al., 1995; Sandercock and Brodie, 2006; Silva et al., 2017). In this regard non-stationary describes having means, variances, and covariances that change over time. Briefly, the root mean square fluctuation of the integrated and detrended data is measured in observation windows of different sizes. The data are then plotted against the size of the window on a log-log scale. The resulting scaling exponents represent the slope of the line, which relates (log) fluctuation to (log) window size (Mendonca et al., 2010). It should be noted that the short-term scaling exponent alpha1 of DFA is applicable in sport-specific settings, since this index requires relative short recording times (DFA-alpha1—default settings with a window width: 4 ≤ *n* ≤ 16 beats). Based on the reasoning of Peng et al. (1995) and Chen et al. (2002) ~200 beats (*n* = N/10) or a 2 min recording window (depending on beats per minute) is needed for a valid calculation to occur. A steady state is not mandatory, as acceptable relationships with DFA-alpha1 and exercise intensity have been demonstrated (Gronwald et al., 2020).

**Figure 1:**
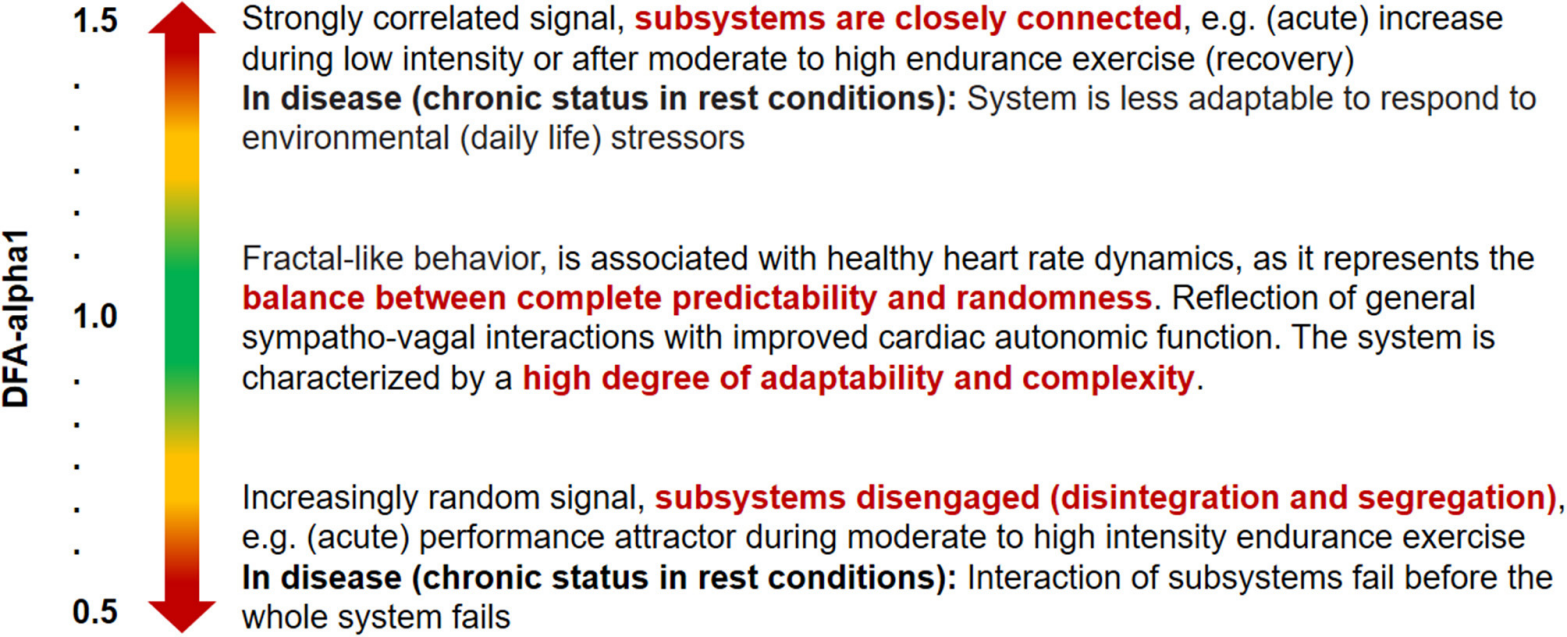
Quantification and qualitative description of acute and chronic correlation properties (short-term scaling exponent alpha1 of DFA) within HR time series during resting conditions and during endurance-type exercise demands (Peng et al., 1995; Goldberger et al., 2002; Lipsitz, 2002; Tulppo et al., 2003; Gronwald et al., 2018).

DFA-alpha1 and its application during resting conditions has already been extensively applied to cardiovascular risk assessment as well as prognosis and prediction of mortality in clinical settings (Platisa and Gal, 2008; Huikuri et al., 2009; Nicolini et al., 2012; Sen and McGill, 2018), and shows that values of DFA-alpha1 that differ from the normal value of ~1.0 (decreasing or increasing) are associated with higher morbidity or worse prognosis regardless of the disease or age group (Huikuri et al., 2009; Sen and McGill, 2018). Both indicate a loss of fractal dynamics and complexity with decreasing values toward a more random (disorganized randomness) or increasing values a more correlated or periodic behavior (de Godoy, 2016). In the context of a homeodynamic regulation approach of the organism, the fractal and complex dynamics of HR time series during resting states may be related to the maintenance of basic stability of controlling systems between order (persistence) and disorder (change) (Yates, 1994; Kauffman, 1995; Iyengar et al., 1996; Makikallio et al., 1999; Billman, 2020). This seems to be a characteristic of complex biological systems under resting conditions to avoid too much order, but also too much chaos. Finally, losing fractal complexity in such control mechanisms results in less adaptability to cope with varied environmental stimuli (Goldberger, 1997; see Figure 1).

Therefore, DFA and its short-term scaling exponent alpha1 were tested in several applied settings and seem also suitable for sport-specific applications including exercise from low to high intensities, non-stationary data, and relatively short recording times for different conditions (Tulppo et al., 2001; Hautala et al., 2003, 2009). In such an application it is rather the qualitative information of organismic regulation during exercise that seems to be important in order to gain new insights from a dose-response perspective and DFA-alpha1 may be taken as an indicator for exercise prescription and intensity distribution monitoring in endurance training (Gronwald et al., 2019a). Therefore, we would like to stress the notion that DFA-alpha1 can serve as a “global parameter” for the whole system and proxy for organismic demands and complex regulation at the intersection of the central and ANS. Although threshold-based concepts and approaches for endurance exercise and training prescription have a strong theoretical basis with various practical applications, the challenges and pitfalls of determining such individual training zones on the basis of subsystem indicators (e.g., blood lactate concentration, respiratory parameters), make it worth considering a more broadened and systemic perspective.

## 3. State of Research in a Nutshell: Correlation Properties of HRV During Endurance Exercise

The state of research of DFA-alpha1 as an indicator for correlation properties of HR time series has been reviewed most recently (Gronwald and Hoos, 2020). In the corresponding studies investigating high intensity and incremental cycling exercise until voluntary exhaustion, the study results demonstrate a general loss of complexity and variability of R-R interval fluctuations with increasing exercise intensity (Hautala et al., 2003; Casties et al., 2006; Platisa and Gal, 2008; Platisa et al., 2008; Karavirta et al., 2009; Blasco-Lafarga et al., 2017; Hottenrott and Hoos, 2017; Gronwald et al., 2019a,b). More precisely, from low to high intensity exercise, DFA-alpha1 indicates a biphasic course. Depending on the resting value (usually around 1.0 and 1.5), constant or moderately increasing values (up to 1.5) have been reported at very low to moderate intensities, and strongly, almost linearly decreasing values down to around 0.3 from moderate to high exercise intensity (Hautala et al., 2003; Casties et al., 2006; Platisa et al., 2008; Karavirta et al., 2009; Blasco-Lafarga et al., 2017; Gronwald et al., 2019a). This behavior indicates an intensity-dependent change of HR dynamics from strongly correlated to uncorrelated/stochastic or anti-correlated behavior due to a notably strong vagal withdrawal and/or sympathetic activation (Platisa and Gal, 2008; Hottenrott and Hoos, 2017) as well as other factors such as possible intracardiac biochemical changes or coupling mechanisms of different physiological subsystems (Casties et al., 2006; Papaioannou et al., 2013).

During prolonged exercise, data is scarce and primarily limited to low intensity (zone 1) walking indicating an increase in DFA-alpha1 (Tulppo et al., 2001; Hautala et al., 2003). The few available studies that investigated a prolonged running and cycling exercise with medium to high (zone 2 to 3) intensity analyzed a strongly decrease in DFA-alpha1 (Hautala et al., 2003; Gronwald et al., 2018, 2019c, 2020) but were kept to a rather short exercise duration. Although preliminary in nature, these data on behavior of DFA-alpha during different kinds of prolonged exercise emphasize the results of the incremental studies mentioned above. Taken together, these findings support the notion that exercise intensity and duration may have an interacting effect on DFA-alpha1 during exercise. It seems that prolonged exercise with low intensity just above rest (e.g., brisk walking) reintegrates and synchronizes subsystems, while prolonged exercise with low to moderate intensity (around LT1/VT1 and the transition range between aerobic and anaerobic threshold) may initiate a disintegrating process, while culminating into progressive segregation of subsystems and mechanization (performance attractor) of the whole system at moderate to high intensity (Gronwald et al., 2019c, 2020). Therefore, DFA-alpha1 seems to show potential in order to describe the internal load not only as a quantitative indicator, but also as a qualitative marker of the regulatory state of the complex organismic system during endurance exercise.

## 4. Correlation Properties of HRV: A New Indicator for Intensity Distribution?

According to the state of research DFA-alpha1 may provide valuable information for monitoring organismic internal load and individualizing endurance exercise and training prescription (Blasco-Lafarga et al., 2017). After a slight rise from rest, DFA-alpha1 falls rapidly with increasing work rates near the aerobic threshold demonstrating the potential usefulness of this parameter for low intensity zone demarcation (Casties et al., 2006; Platisa et al., 2008; Gronwald et al., 2019a). The rate of change per workrate elevation seems highest near the aerobic threshold and since it is a complexity index of overall organismic demands (Gronwald et al., 2019a), no curve calibration or normalization is needed. Studies by Casties et al. (2006), Platisa et al. (2008), Blasco-Lafarga et al. (2017), and Gronwald et al. (2019a) all indicate a clear decline in interbeat correlation properties with high work rates with values resembling white noise behavior at VO_2_ levels historically in the realm between aerobic threshold (LT1, VT1) and anaerobic threshold (LT2, VT2). Within the context of different training models (e.g., polarized, pyramidal, or threshold training-intensity distribution; Seiler and Kjerland, 2006; Stöggl and Sperlich, 2015; Bourgois et al., 2019) this may offer a new perspective, based on systemic autonomic regulation, to determine exercise and training zones for endurance-type sports guided by DFA-alpha1. An intensity distribution limited to the region around the aerobic threshold could be implemented on the basis of DFA-alpha1 values between a self-similar (fractal) HR time series with high complexity (DFA-alpha1: 1.0) and a completely random regulation dynamic in the HR time series with low complexity (DFA-alpha1: 0.5), thus defining a zone 1 threshold of ~0.75. In this regard, it seems possible to distinguish between the above mentioned two organismic states based on ANS regulation; (1) integration and synchronization of subsystems at low exercise intensity and (2) progressive segregation and mechanization of subsystems at high exercise intensity; supported by the behavior of counter regulation (reintegration) with high DFA-alpha1 values during passive or active recovery (Casties et al., 2006; Gronwald et al., 2019b). Despite the application of 0.75 as a cut-off value for survival curves and mortality rate assessment according to resting conditions in clinical settings (Huikuri et al., 2000), an exact value of DFA-alpha1 for passing a low intensity threshold cannot be determined at present. However, it can be stated that values of 0.5 are in any case too low (Gronwald et al., 2019a).

**Figure 2:**
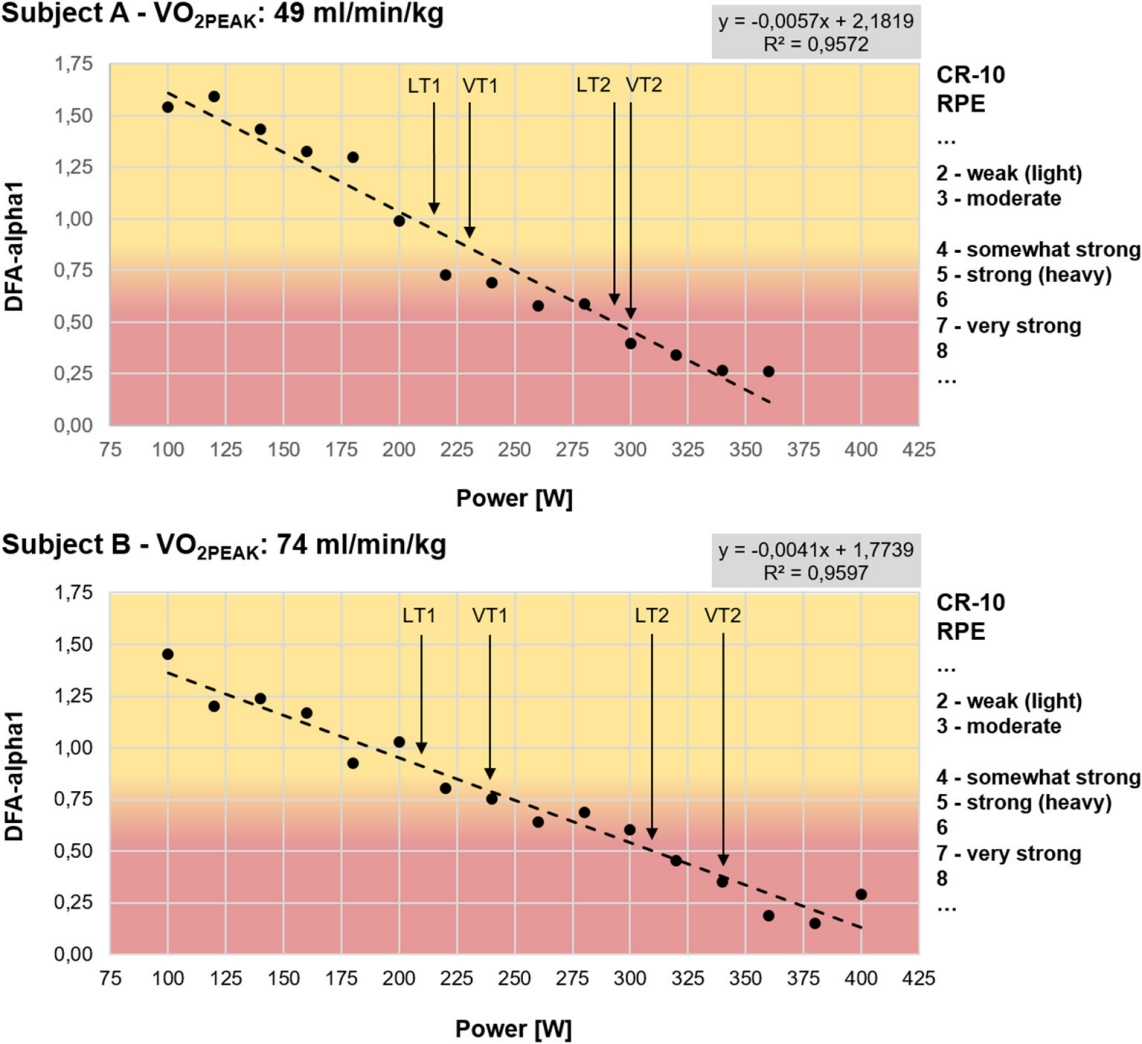
Test results of two subjects during a graded cycling exercise test until voluntary exhaustion with additional information to rating of perceived exertion (RPE) according to CR-10 scale from Borg (1982), lactate and ventilatory thresholds and a colored transition zone around DFA-alpha1 of 0.75 (start: 100W, increment: 20W, stage duration: 3 min). Subject A with a maximum achieved wattage of 360W and VO_2PEAK_ of 49 ml/min/kg (LT1: 217W, VT1: 230W, LT2: 294W, VT2: 300W); Subject B with a maximum achieved wattage of 400W and VO_2PEAK_ of 74 ml/min/kg (LT1: 210W, VT1: 240W, LT2: 310W, VT2: 340W). Lactate thresholds were determined according to Dickhuth et al. (1999) and ventilatory thresholds were determined according to Gaskill et al. (2001) and Binder et al. (2008); DFA-alpha1: short-term scaling exponent alpha1 of detrended fluctuation analysis, VO_2PEAK_: maximum oxygen uptake achieved in the graded exercise test, LT1: first lactate threshold, LT2: second lactate threshold, VT1: first ventilatory threshold, VT2: second ventilatory threshold.

To this date, only limited information is available regarding DFA-alpha1 behavior specifically in relation to different established threshold-based concepts. A careful look at the incremental ramp study by Gronwald et al. (2019a) shows that at an average blood lactate of 2.87 mmol/l, subjects had a DFA-alpha1 of 0.49, a value consistent with random fluctuations (white noise). In addition, analysis of two subjects from that study with disparate VO_2PEAK_ levels is consistent with DFA-alpha1 transition to a less correlated state just around the transition between aerobic and anaerobic threshold (see Figure 2). Despite the limitation of 2 min windows of analysis on each stage of 3 min, which does not occur under steady state VO_2_ conditions (despite wattage being stable), the two individuals with markedly disparate measured aerobic fitness show transitions of DFA-alpha1 values of 0.75 at similar relative work rates (~60–70% of maximum wattage). Therefore, although suggestive as a marker for zone 1, further studies using artifact-free longer, steady state conditions are warranted for estimation of potential DFA-alpha1 cut-off values especially with the focus on a sensitive low intensity range around aerobic threshold (with heterogeneous populations according to performance level, age, and sex), may be assessed by time-varying analysis methods. DFA-alpha1 has a good dynamic behavior around that intensity range and therefore is a promising index to study for those purposes.

In this regard, in a recent case report by Rogers (2020), it was shown that DFA-alpha1 even reached values of 0.75 near the VT1 and had further declines to 0.5 (white noise) at 20 watts above VT1. In that study, through the use of beta blockade, it was shown that the DFA-alpha1 decline was related to cycling power and ventilation rate rather than absolute HR, supporting the organismic demand hypothesis. It is important to note that this study utilized 5 min constant power cycling intervals as opposed to shorter incremental ramps. Given the steady rise in VO_2_ during an incremental ramp, direct comparison of the results may be misleading. Therefore, for a research and practical perspective, further studies are mandatory to confirm these first indications on the basis of the current state of research and to open the way for the use of exercise and training prescription in endurance-type sports.

**Figure 3:**
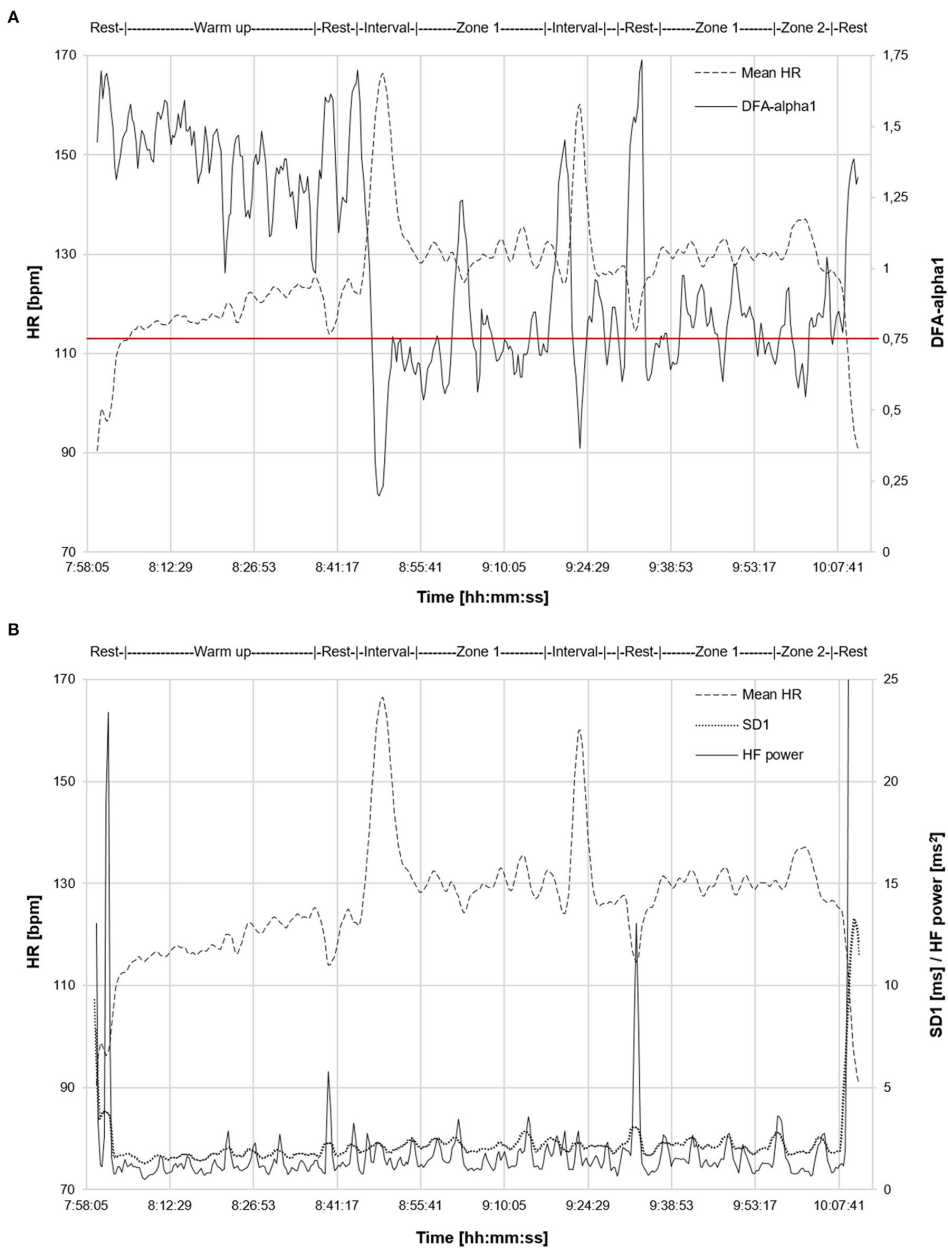
Time-varying analysis (window width: 120 s, grid interval: 20 s) of a 2 h exercise session from a master cyclist with a previously measured VT1 at HR 130 bpm by gas exchange. Data recorded with a commercial chest belt monitor (Suunto Movesense, Vantaa, Finland) and processed in Kubios HRV Software (Version 3.4.1) using automatic correction method (artifact percentage below 0.5%). **(A)** Mean HR and DFA-alpha1 are plotted over time. **(B)** Mean HR, SD1 and HF power are plotted over time. Intensity range of exercise (warm up, brief rest, zone 1, zone 2 and high intensity intervals) indicated on graph. DFA-alpha1: short-term scaling exponent alpha1 of detrended fluctuation analysis, VT1: first ventilatory threshold, SD1: standard deviation 1 from Poincaré plot HRV analysis (short-term index), HF power: high frequency power of frequency-domain HRV analysis.

If future investigation confirms that a specific value of DFA-alpha1 is associated with the VT1 transition, observation of this index in real time during activity, or after completion may be useful to both athletes and coaches. A brief example of a suggested use case scenario is presented in Figure 3. A master cyclist (measured VT1 at HR 130 bpm by gas exchange) interested in verifying that training is properly polarized, performed a 2 h exercise session. DFA-alpha1 was between 1.4 and 1.0 (consistent with very low intensity) during a 40 min warm up and continued at or above the proposed 0.75 limit as long as heart rate remained below VT1 limits. There was moderate DFA-alpha1 decline during the zone 2 portion and substantial decline of DFA-alpha1 to values below 0.4 during the two high intensity intervals. In contrast, although both SD1 and HF power changed from rest to exercise states, there was little dynamic change over the course of the polarized session with no differentiation between warm up, zone 1, zone 2, nor high intensity intervals.

## 5. Limitations and Future Research Directions

Despite the promise of this approach, a few possible limitations should not be overlooked. We are aware that endurance exercise physiology is complex, with many influencing factors, and can‘t be broken down into a single key measure. Therefore, we recommend to include such an indicator of systemic regulation based on ANS in the diagnostic and monitoring routine (such as cycling power, heart rate), to gain additional insights to well-established threshold-based concepts for intensity zone distribution in endurance-type sports for zone 1 to zone 2 transition (LT1/VT1). In addition, although DFA-alpha1 does continue to fall at higher intensities including zone 2 to zone 3 transition, it does so at a much lower rate depending on subjects training state (Platisa et al., 2008). Given this restricted dynamic range at high work rates, it is not ideally suited as a measure of a high intensity threshold. Alternate methods for zone 2 to zone 3 transition are available including measurement of the RCP/LT2 by means of gas exchange, lactate testing or simply by functional threshold power (FTP) interval testing (Meyer et al., 2005; Binder et al., 2008; Beneke et al., 2011; Hofmann and Tschakert, 2017; Lillo-Beviá et al., 2019). Besides the possible limitations stated by Gronwald et al. (2019c) and Silva et al. (2017) regarding an unclear detailed physiologic interpretation of DFA-alpha1 and a possible influence of spontaneous breathing during exercise enabling physiologic coupling processes, some caution in HRV analysis interpretation during moderate to high intensity exercise may be needed if artifacts are present (Rincon Soler et al., 2018). Giles and Draper (2018) showed an almost 5% artifact rate at high exercise intensities that lead to substantial bias in both frequency-domain and non-linear indexes despite correction methods, for example from Kubios HRV Software (Version 2.2). Therefore, as exercise intensity rises, so does the likelihood for artifacts which potentially can blunt the drop in HRV correlation properties. Giles and Draper (2018) further recommended using near artifact free tracings, stating artifact presence and which correction methods are used in reporting results of HRV analysis. This potential shift in non-linear complexity change may lead to difficulty comparing study results of DFA-alpha1 behavior.

We would also like to suggest future areas of study to better define the potential benefits, pitfalls and unknowns in using this index for exercise intensity modulation. As previously stated, artifact correction may play a role in creating index bias and needs to be explored in detail. Other interesting future issues are cardiovascular drift and hydration status, as well as heat and emotional stress (Coyle, 1998; Coyle and González-Alonso, 2001; Souza et al., 2019; Macartney et al., 2020). It is certainly possible that depending on the testing conditions (ambient temperature, presence of a cooling fan, anxiety level), an index based on ANS regulation may yield disparate results depending on variables not usually thought of. Most gas exchange cycling ramps are done without air flow cooling which may or may not transfer similar results to road riding conditions. Since many prior studies examining DFA-alpha1 behavior during exercise are based on VO_2_ or power output, future work could also further elucidate the general relationship between DFA-alpha1 and heart rate (Platisa et al., 2008; Gronwald et al., 2019a) e.g., by systematically incorporating the influence of ramp or graded exercise protocol. As opposed to power output/VO_2_, heart rate would be a more direct surrogate for added autonomic influence. Other topics for study may include but are not limited to the effects of age, sex, prior fitness status, and cardiac risk factors. Given that many of the subjects examined thus far are male, healthy and fit, extension of index usefulness to other populations is very important. In addition, different methodological influences should be further investigated (e.g., window lengths for DFA-alpha1 calculation, moving window for averaging, signal-to-noise ratio, test protocol—increment and duration, DFA-alpha1 breakpoint values). Finally, if these questions can be answered, DFA analysis of HRV may represent a valuable modality to view physiologic regulation during endurance exercise, and it may be useful for commercial wearable manufacturers in combination with other metrics of internal and external load for diagnostics and monitoring in endurance exercise and/or training.

## Data Availability Statement

All datasets presented in this perspective are included in the article.

## Ethics Statement

The studies involving human participants were reviewed and approved by University Clinic of Halle (Saale), Medical Faculty of Martin Luther University of Halle-Wittenberg. The participants provided their written informed consent to participate in this study.

## Author Contributions

TG and BR drafted the article. All authors have conceived the perspective. All authors revised it critically for important intellectual content, final approval of the version to be published, and accountability for all aspects of the work.

## Conflict of Interest

The authors declare that the research was conducted in the absence of any commercial or financial relationships that could be construed as a potential conflict of interest.
